# Do households prefer to move up or down the urban hierarchy during an economic crisis?

**DOI:** 10.1007/s10109-021-00353-7

**Published:** 2021-05-22

**Authors:** Eveline S. van Leeuwen, Viktor A. Venhorst

**Affiliations:** 1 Urban Economics Group, Wageningen University, Wageningen, the Netherlands; 2 Faculty of Spatial Sciences, URSI, University of Groningen, Groningen, the Netherlands

**Keywords:** Global financial crisis, Recession, Urban–rural hierarchy, Willingness-to-move, Motive for mobility, Internal migration

## Abstract

In this paper, we investigate the relationship between adverse economic circumstances and the desire of Dutch households to move up or down the urban hierarchy. We apply three consecutive waves of the Dutch Housing Demand Survey (WoON) in a repeated cross-section setting, with data collected at the time of the Global Financial Crisis (GFC) and its aftermath. We find that households desire to move down the urban hierarchy during the volatile and uncertain periods following the GFC. This is a surprising result, given that urban areas are generally considered more opportunity rich. In order to uncover the mechanisms driving this result, we considered the impact of the economic circumstances on the general willingness to move and on the underlying motives. We find that willingness to move increased when the adverse economic consequences of the GFC hit Dutch households. Further, it appears that this willingness to move is only partially related to work. Besides work, desires to move for health, education, vicinity to family and friends, and reasons related to the dwelling, also become more prevalent during the aftermath of the GFC as well. This heterogeneity in impacts and consequences for household desired mobility serves to explain some of the mixed results in the literature, and generates lessons for current and future crises such as the Covid-19 pandemic.

## Introduction

Discrepancies between the actual location of a household and the location that is desired can occur at any time. However, these discrepancies can be exacerbated during an economic crisis. On the one hand, housing demand is shocked, in the sense that a household may no longer find itself in a favourable location. For example, particular regions may be hit relatively hard, or, as observed more recently during the Covid-19 crisis, the actual dwelling may prove unsuitable to sustain prolonged periods at home. On the other hand, the crisis can be such that it becomes more difficult for the household to effectuate its demand due to, for example, financial constraints. In times of crisis, spatial mobility can be thought of as an equilibrating force, literally enabling individuals to re-position themselves in the economy.

In the wake of the Global Financial Crisis (GFC) from mid-2007 until early 2009, there has been considerable attention in the literature to what extent workers and households were willing and able to escape from adverse local economic circumstances through migration. Recent studies suggest that migration failed to exert its role in spatially (re-) aligning households and opportunities. The limited availability of credit and housing to those entering the labour market, versus the likely reluctance of others to leave in uncertain times, may serve to lower spatial mobility in times when relocation could help to improve personal and regional outcomes.

Spatial mobility is generally higher in the USA than in Europe (Molloy et al. 2011). However, Cooke (2013) observes that, in the USA, and contrary to most European countries, migration rates have been declining steadily for the last few decades. The GFC did not change that trend very much. What did prove to be important was an increase in dual-worker couples, increased household indebtedness, and the widespread rise of information and communication technologies (ICTs). Coulter et al. (2016) conclude that, in a context of linked lives, constraints to mobility may result in a situation of unmet residential desires, which in turn could lead to progressively worse outcomes in other life domains. The GFC was rooted in the housing market and has directly or indirectly influenced the lives of many households. It led to the tightening of funding available for mortgages, as well as lower house construction rates (De Groot et al. 2011). With subsequent economic improvements, in many urban environments the housing markets started booming again, weakening the opportunities of new cohorts to enter the housing market (De Jong et al. 2016), in particular in such urban areas. Thus, as Coulter et al. (2016) note, the GFC can be thought of as re-aligning the positions of groups in different life course stages in society.

Insofar as a crisis leads to residential lock-in, it may very well also lead to what can be referred to as residential dissonance, as Kamruzzaman et al. (2013) point out. There might be a relationship between economic crises and the extent to which people find themselves in a residential position, which is no longer desired. In this paper, we will argue, firstly, that it is vital to consider that individual, household or even regional economic constraints may be such that the desired move is impossible. As revealed moves represent successes in residential mobility only, we shall rely on stated intentions instead. The literature on the relationship between economic crises and migration typically relies on actual moves and migrations, whereas, in particular in times of economic hardship we must consider the fact that a willingness to move is not always effectuated, resulting in a discrepancy between wish and reality. We stress here that, in our view, these discrepancies should be understood in a wide sense. It could refer to access to jobs, the size of the dwelling that is no longer deemed fitting, the vicinity of support networks of friends and family or the wider characteristics of the neighbourhood. An economic crisis can take effect on many domains of life.

Secondly, we will therefore conceptualise this discrepancy in this paper along the urban–rural axis, as the two extremes represent many of the elements of discrepancies as potentially felt by households. It is conceivable that, in times of crisis, risk-averse households would prefer destinations further up the urban hierarchy (Plane et al. 2005), i.e. towards the Central-Urban areas, where more job opportunities or other support networks present themselves. Others might prefer to move down the urban hierarchy, to areas where house size and outdoor space are easier to acquire, thereby facilitating flexibility in how households can combine labour supply and other domains of life. It is also conceivable that, depending on the nature and the personal impact of a crisis, in the event that a move is deemed necessary, locations similar to the current one are preferred.

The main research question guiding our analysis is therefore: to what extent is there a relationship between regional economic circumstances and the desire of households to move up or down the urban hierarchy during an economic crisis? In order to investigate this, we first analyse the willingness to move: is it indeed the case that adverse economic circumstances make households less willing to move as the earlier literature on revealed migration seems to suggest? If indeed there is an effect, we furthermore need to understand in what way the crisis fuels this desire for location change: what is the motive for the desired move, and do motives depend on the economic circumstances? Is it the quest for a location with improved access to jobs, or is it driven by other factors such as the dwelling, health or the vicinity of family and friends? This will feed into our main analysis of the relationship between economic crises and the desire to move up or down the urban hierarchy. Is a move up or down the urban hierarchy indeed fuelled by the changing economic circumstances and considerations, or do other mechanism emerge?

This study adds to the literature by deepening our understanding of the impacts of economic crises on household spatial mobility. The paper does so by looking at stated intentions, which provide a fuller picture of desired change than looking at successful moves only. Furthermore, the paper does so by applying a wide conceptualisation of discrepancies in actual and desired location (i.e. across the urban–rural axis, and all these locations have to offer) considering all motives for mobility (i.e. not just work-related motives). To this end, we use three consecutive waves from the Dutch WoON/Housing Demand Survey (2008–2015), which provides us with a repeated cross-section of Dutch households sampled during the onset of the GFC, and in the recessionary years thereafter.

## Conceptualising mobility during economic crises

### Stated and revealed preference

Rushton (1969) postulates that a proper understanding of spatial behaviour should be based on the identification of the true spatial preferences of individuals, rather than a description of the revealed behavioural patterns. The individual is deemed perfectly capable of producing a relatively stable ranking of all conceivable spatial opportunities as presented at a given time. However, revealed spatial behaviour is the result of the application of these preferences to a choice set which may be constrained by the actual circumstances, including the spatial context, personal circumstances and economic shocks. Is it then the preference structure that is observed, or rather the spatial behaviour that is generated by the actual context? When analysing early retirees migrating towards rural Wales, Stockdale (2014) demonstrates that preferences and images play a role in migration events, but also that the move is usually accompanied by specific life events, such as retirement or children leaving the parental home. Interestingly, she notes that the property that is eventually chosen is often considered to be somewhat beyond the control of the migrant: it depends on what happens to be available.

Psychological theories of reasoned action (Ajzen and Fishbein 1980) suggest that intentions lead to behaviours if attitudes, norms and capabilities all line up. Stated intentions or preferences therefore do not always match revealed behaviour. It is tempting to think of these mismatches as an error or as evidence of a lack of perfect foresight on the part of the decision maker. Manski (1990) argues that even under the classic model of the homo economicus discrepancies can occur: any difference between the actual (i.e. revealed) behaviour, and an intention which was stated earlier, can be viewed as evidence of new relevant information becoming available, such as a change in the situation of the decision maker or a change in the circumstances on the housing market. In particular in studies where intentions were measured in straightforward yes/no questions, the respondent is not made aware of the option to include uncertainty or probable future events into their reasoning. The time frame considered is also of critical importance in reconciling intentions and actual outcomes: how long is the adjustment time? The question is then not *who* has made an error or misstated their intentions. Rather, we can ask *when* circumstances change to such an extent that the respondent considers themselves to be no longer in a desired situation. In this paper, we focus on economic crises as such a change in circumstances, leading to a desired change (i.e., a willingness to move and, more specifically, a willingness to move up or down the urban hierarchy).

The literature, which we discuss in the next section, on the other hand, predominantly focusses on actual residential outcomes in light of earlier expectations or stated intentions. A discrepancy is then considered an outcome, moving or staying, that differs from what was stated before. This approach carries the risk of confounding the role of changes in circumstances, leading to current discrepancies, and the demonstrated (in-)ability to effectuate the desired housing demand. Some households are observed in situations that deviate from their earlier intentions because they were able to respond to intervening changes in circumstances. Others are observed in situations that are similar to what they intended to do, but it is not a priori clear whether this is the result of a lack of new circumstances or an inability to respond. Reversing this approach, i.e. analysing current desires in the light of current actual residential situations has the advantage of avoiding these issues. Therefore, rather than using the more classic approach of analysing longer distance actual migrations, we follow Feijten et al. (2008) and Schwanen and Mokhtarian (2004) in analysing discrepancies between currently stated desires and the current residential situation: compared to where they live now, do people desire to move up or down the urban hierarchy?

### Economic crises and the desire to move up or down the urban hierarchy

It has been noted that people are reluctant to opt for long-term solutions (such as migration) in response to problems that they perceive as cyclical or temporary in nature (Coulombe 2006). Following Kahneman and Tversky (1979), Clark and Lisowski (2017a, b) note how endowments would lead risk averse individuals to remain in areas even if these areas are hit by adverse economic circumstances. Bounded rationality, limited information, and the current use value lead together to a high valuation of the status quo, in particular in the case of decisions that are neither taken often nor lightly, such as migration. Furthermore, uncertainty increases in an economic crisis and therefore the likelihood of an unexpected or sudden move in order to adjust to a new economic reality becomes then even less likely (Clark and Lisowski 2017a).

The evidence in the literature on the relationship between the GFC and revealed migration, in terms of the likelihood to move and in terms of destination choice in times of crisis, is mixed. Although long distance migration did not increase, Lim (2017) notes that labour market participation was associated with a push into short distance moves out of markets that were badly hit by the GFC. Others see immobility as a response to the GFC, either as the result of lock-in (Modestino and Dennett 2013) or as a coping strategy (Preece 2018). In a study of the four largest cities in Spain, Bayona-i-Carrasco et al. (2017) describe how foreign migration flows were altered due to the GFC, whereas migration decisions by Spanish nationals did not change very strongly in spite of high unemployment rates. The authors detected that there appeared to be a shift in the type of destination that was sought. Suburban areas, popular during the economic boom period, were less favoured afterwards, compared to the urban and inner-ring towns which were losing population to a lesser extent. As in the case of the USA (Cooke 2013), migration may have fallen during the crisis, but at least partially for reasons seemingly unrelated to the crisis. Cooke (2013) pointed towards household and employment characteristics. In the Spanish case, the main driving force appears to be structural change in population composition as a result of aging (Bayona-i-Carrasco et al. 2017). Remoundou et al. (2016) note, for the case of Athens, on the basis of a stated preference experiment, an increased likelihood to leave Athens for the suburbs. According to the respondents, this change in stated preferences was crisis-led.

These results therefore suggest a diverse impact of economic crises on (desired) spatial mobility. In part, this could be related to the factual impact of the crisis on individual households: it may very well be that it was not (just) individual employment that was affected, but, rather, housing options or the position of family and friends. Therefore, understanding a change in desired location must go hand-in-hand with a deeper understanding of why people wish to relocate in response to the crisis.

Motives for moving have received quite some attention in the literature (Coulter and Scott 2015; Morrison and Clark 2011; Niedomysl 2011; Thomas et al. 2019). We will return to the main findings of these studies when we compare these with descriptive statistics from our sample. For now, it is important to note that these studies typically demonstrate that migration distance is associated with motive (longer distances for work and study, shorter distances for pure residential moves, moving towards family and friends across the whole distance spectrum) and that the likelihood of actually engaging in a move depends on how specific and urgent the motive is. Furthermore, motives for migration differ between individuals in varying phases in the life-course. This signals that, if indeed economic crises impact on other motives to move, besides work, we can expect to see a diverse mobility response of households faced with the adverse effects of a crisis. The adjustment to the crisis is then not just about moving towards more urban regions with good economic opportunities.

### Key independent variables in this analysis

As will be demonstrated in Figs. 3 and 4 further down, the GFC affected the Dutch economy in distinct phases: initial stagnation, (delayed) impact and subsequent recovery. House prices responded with some delay to the onset of the GFC, first with stagnation and then decline. Conversely, the regional unemployment rate increased substantially during the crisis. From 2013, onwards house prices and regional unemployment rates showed the first signs of recovery. Furthermore, there was regional variation in the strength of these fluctuations. Therefore, we proceed to capture the overall impact of the GFC using period dummies. An impact which, given the earlier discussion on the heterogeneity in (desired) household response to crisis situations, is necessarily measured in this general way. We use these dummies in combination with two variables which capture the regional dynamics in house prices (based on transactions) and unemployment rates relative to the Dutch national average, and which are calculated as relative running indices.^1^ These variables indicate whether specific regional impacts of the GFC, over and above the general patterns, have further consequences for desired residential mobility.

Using these variables, we investigate the relationship between economic circumstances and the desire to move up or down the urban hierarchy, considering also the willingness-to-move and motives. In doing so, we have to be mindful of potential endogeneity. Firstly, reverse causality might impact on the findings, in particular as far as the development in house prices is concerned. If many people desire to leave a particular region, this might depress the house price level. For this reason, we have opted to measure house prices at the relatively high spatial scale of NUTS2 (Dutch provinces). At this level, there should be less of an impact of micro-preferences on the local housing market situation. For unemployment, endogeneity issues play potentially less of a role, in particular because we control for individual employment status. It seems unlikely that people will either exit their job or engage in finding work as a result of a willingness to move. Only after a migration has been successfully planned or even executed, one would expect to see such steps. Secondly, it could be that other regional circumstances simultaneously influence the house prices or unemployment rates as well as the willingness to move up or down the urban hierarchy. Thirdly, the outcomes might be driven by degree of selection of households into regions (i.e. their current residence) and their degree of mobility. Including mobility motives helps mitigate issues with selection on unobservables and attitudes. Constraints to mobility are not always captured by observable characteristics such as household size or age, but rather might be driven by the general outlook on, and priorities in, life. Motives may serve to capture those. Furthermore, we apply a rich set of control variables at the household level, regional fixed effects and clustered standard errors at the regional level to mitigate these potential problems. The theoretical notions underpinning these control variables will be introduced in the next section.

### Control variables

The literature provides us with a number of essential insights into mobility processes in general, necessitating the addition of a number of control variables. De Jong et al. (2016) discuss how age groups differ in their migration patterns up and down the Dutch urban hierarchy. Even though the patterns are not exclusive for them, younger migrants tend to move up the urban hierarchy, whereas older migrants tend to move down towards medium sized towns which are quieter but still provide ample care. The authors do not focus on the housing market crisis as such, but they do note that constraints and mismatches between demand and supply could result from relative size differences of different birth cohorts simultaneously moving up and down the urban hierarchy. This could show up as temporal variations in discrepancies between supply and demand, but their cause should thus not necessarily be sought in current period-specific shocks. Whisler et al. (2008) also stress the importance of properly accounting for the different demands of the varying age groups in analysing migration patterns, to the point where they conclude that composite indices of the quality of life of particular destinations that do not account for age-related differences in quality-of-life factors, are virtually “meaningless” (p. 89).

Clark and Davies Withers (2007) allude to another form of heterogeneity as they demonstrate that the straightforward linkage between employment and long-distance moves, and housing mobility and short distance moves, is increasingly an over-simplification in the light of new family formation processes. Moves are found to be increasingly diverse, gendered, sequential and unintentional. In this light, Coulter et al. (2016) reiterate that age is generally a poor proxy for life events, given the increased heterogeneity in life course trajectories and the timing of key life events. Schwanen and Mokhtarian (2004) find that household composition is important in predicting the occurrence of residential discrepancies, in particular for singles with a preference for urban living and large families with children, with a preference for more rural and spacious areas.

Therefore individual, household and contextual factors may interact to retain households in their place. Coulter (2013a, b) shows that older households, and those with larger life-course ties and commitments, have a higher likelihood of abandoning a previously intended move. Coulter (2013a, b) also reports such a relationship with income and ethnicity. The GFC may have inhibited moves by home owners in particular. The author notes that these processes can contribute to segregation and social inequality. Extending this point, Coulter and Van Ham (2013) uncover some interesting risk factors: existing couples are more likely than newly formed ones to abandon mobility plans. They also note that higher income may prevent the occurrence of spells in undesired states.

With the greater availability of longitudinal micro level datasets, the question of the operationalisation of the different dimensions of time comes more and more to the forefront. Feijten et al. (2008) look into the effect of gaining experience of certain locations, which is found to increase the likelihood of either returning to that location, or selecting a similar location (in the case of rural experience). The authors note that, in many of the instances in which return migration was on the cards, the presence of a social network in the destination region was driving the behaviour. Yet, with increasing duration at a certain type of location, the likelihood of moving to a similar location increases. Furthermore, the authors control for period-specific shocks to the housing market.

In the literature, there is therefore ample evidence for general period-specific differences in accessibility of the housing market, which in turn suggests a higher likelihood for some households to be involuntarily stuck in a particular situation, or at least experiencing a discrepancy between their current and desired residential situation. What has become additionally clear is that the housing market positions of older cohorts are instrumental in preventing the fulfilment of housing desires, which may also affect younger households. We will therefore consider age, tenure and the type of residence at the existing residential location. As discussed above, controlling for actual life course states is found to be important, next to controlling for age, as life histories become more and more diverse. The household situation, including the presence of young children, whether or not a partner is present and whether the partner is working, is therefore also included. We also control for ethnicity, labour market status and household disposable income.

To conclude, we are particularly interested to see how the desire to move up or down the urban hierarchy relates to the urbanity of the current residential neighbourhood, specifically during an economic crisis. The desire to live in a neighbourhood with more urban or more rural characteristics, compared to the current location, may be fuelled by a number of potentially confounding factors. In order to identify the extent to which the urban–rural hierarchy itself is important, we control for a number of related issues. Firstly, we control for whether the household intents to leave the current municipality, or rather envisages to move within the current municipality. It is likely that within a given municipality, the destination neighbourhood is similar to the current neighbourhood. Second, we control for the characteristics of the current and desired dwelling, as particular dwelling types are not ubiquitous. We include regional fixed effects for the current residential region in order to account for any other unmeasured push factors.

In the next section, we present descriptive statistics concerning (post-)crisis mobility. Following that, we construct our models of the impact of the GFC on willingness to move, geographic mobility motive and the extent to which respondents desire to move up or down the urban hierarchy.

## Dataset, variables and descriptive statistics

### The Dutch housing demand survey dataset

To gain more insight into the impact of the GFC on the desire of households to move up or down the urban hierarchy, we make us of the Dutch WoON (Housing Demand Survey) dataset. This survey has been carried out every three years for the last two decades by Statistics Netherlands in cooperation with the Ministry of Internal Affairs. Every wave, a new cross-section of around 70,000 respondents is interviewed about their residential preferences, willingness to move and their household characteristics. It takes the respondents between 20 and 60 min to answer all questions. This information is enriched by Statistics Netherlands with information from the municipal administration and the national tax authority. Even though the survey is, for the most part, identical in each wave, some questions are included in only a few waves. We make use of the 2009, 2012 and 2015 waves. Our initial pooled sample consists of a total of 183,897 respondents, of whom around 27% express the willingness to move within the next two years. For these potential movers, we analyse the intended direction of the move: up or down the urban hierarchy, relative to remaining in a similar area. This spatial mobility may or may not actually take place. Hence, and contrary to many other studies, there is no cut off or minimal distance in order for a move to qualify as mobility: the distance of an intended move is not yet known. Furthermore, given the heterogeneity in mobility outcomes as reported in the literature, we necessarily employ a broader conceptualisation of (desired) mobility. Our sample therefore also contains individuals who are eyeing only very short distance moves, which usually have a much stronger residential objective, rather than an economic one (Thomas et al. 2019).

### Up or down the urban hierarchy

**Fig. 1:**
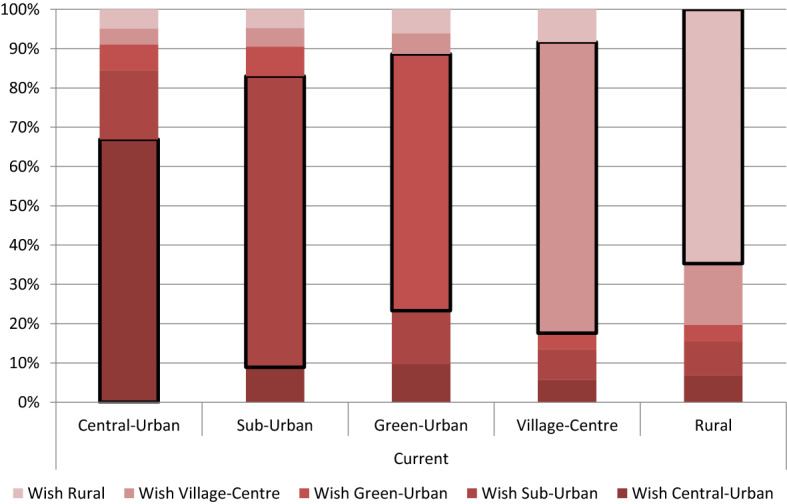
Preferred destination according to current type of neighbourhood (outlined categories mark respondents who prefer destinations which are similar to the current neighbourhood). Source: WoON 2008–2015; Statistics Netherlands

We operationalise residence-related discrepancy by comparing the degree of urbanity of the current and the desired neighbourhood. The typology used in our WoON data distinguishes between Central-Urban, Sub-Urban, Green-Urban or town, Village-Centre and rural. Based on the respondent’s address, Statistics Netherlands derived the current type of neighbourhood. For those willing to move, the urbanity of the preferred destination has been either asked directly, or imputed from questions about the desired size of the municipality, distance from Central-Urban, type of dwelling in the neighbourhood, and building period.^2^ To establish the intended direction of the move, we compared a respondent’s current type of neighbourhood with the one that is desired. If the desired neighbourhood is more urban than the current one, e.g. if a person is currently living in a Sub-Urban neighbourhood and would like to move to the Central-Urban, this is translated into a desired move “up” the urban–rural hierarchy. Consequently, this means that people who are currently living in the Central-Urban cannot move further up the hierarchy, only down; and people currently living in rural neighbourhoods cannot move further down. When the type of neighbourhood is similar, they prefer the “same”. Across our sample, we find that most people prefer moving into the same type of neighbourhood. About 13% of the respondents, living in Sub-Urban areas or lower, prefer to move up the urban hierarchy. Conversely, 17% of the respondents living in Village-Centre and further up, prefer to move down. Around 73% of the sample prefers to move into a similar neighbourhood.^3^ Figure 1 shows the differences in destination desired, by those with a willingness to move, according to the degree of urbanity of the current environment. The figure confirms that, in general, people desire to move into similar neighbourhoods, but that there is also a substantial latent demand for change of up to a third of the residents in particular areas. Also, when a different type of neighbourhood is desired, it is not necessarily the next type up or down the hierarchy that people desire.

### Willingness to move

**Fig. 2:**
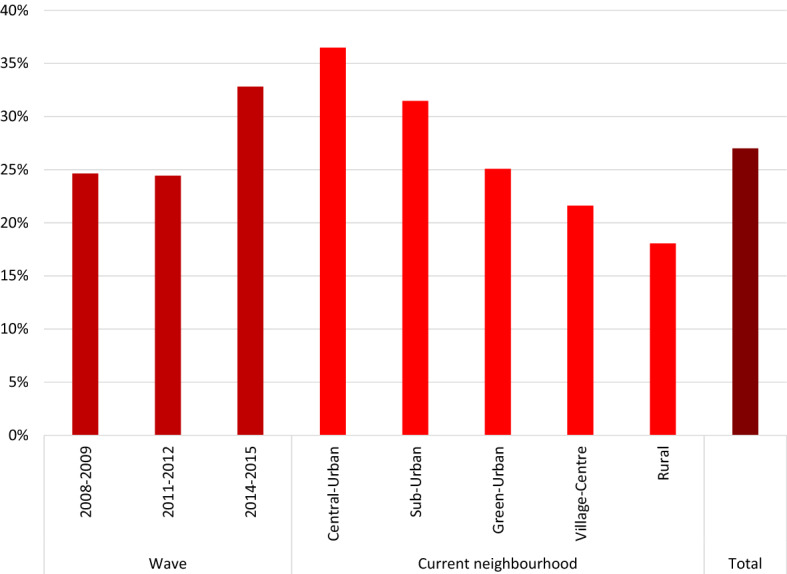
Share of respondents who want to move by survey wave and by their current neighbourhood type. Source: WoON 2008–2015; Statistics Netherlands

For *Willingness to move*, we used the question “Do you want to move within two years?” as a filter. Respondents who answered “Definitely”; “Possibly”; “Yes, but cannot find anything” are considered willing to move. Others are considered not willing to move. The resulting dummy is the dependent variable for the first analysis. In the subsequent steps, we focus on those willing to move to analyse moving motives and the preference for moving up or down the urban hierarchy. As described earlier, the total sample of the three waves between 2009 and 2015 consists of 183,897 respondents of whom 27% wants to (perhaps) move within the next two years. Figure 2 shows that during the first two waves the percentage of those willing to move is around 24%, only to increase to around 33% at the peak of post-GFC unemployment. This suggests that during the height of the actual GFC in 2007–2009, the willingness to move was in fact lower, compared to the years that followed the GFC. The fall-out in terms of housing price fluctuations and unemployment hit most households with a delay. Those that are willing to move are not evenly spread over the country: people living in cities are more likely to want to move within the next 2 years, whereas latent demand for change seems lower in the more rural areas. Figure 2 shows how 36% of the people currently living in a Central-Urban are willing to move compared to only 18% of the people living in a rural neighbourhood.

### Motive for mobility

The willingness to move can be driven by different motives. In the questionnaire, those respondents who indicated a willingness to move were subsequently asked to state the motives for that move. We use the following six motives in our analysis (respondents were able to select more than one): motives related to health or the need for care, move to study, move for work reasons, move for reasons related to the dwelling, move for reasons related to the neighbourhood and, finally, moving closer to family and friends. As can be seen in Table 1, the most frequently selected motives in the pooled sample are related to the current dwelling (about 40%), with characteristics of the neighbourhood a distant second (about 25% indicated this). Nearly 15% of our respondents indicate that they move related to health issues. Work is mentioned by 12% of the respondents, followed by moving closer to family and friends (9%) and study (3%).

**Table 1:** Motives for prospective move in percent of those willing to move by survey wave

	2008–2009 (%)	2011–2012 (%)	2014–2015 (%)	Pooled sample (%)
Health	12	13	19	15
Study	3	3	4	3
Work	10	11	13	12
Dwelling	39	38	42	40
Neighbourhood	25	24	25	25
Family and friends	8	9	11	9

*Source*: WoON 2008–2015; Statistics Netherlands. *N* = 37,389. Respondents (which include only those who indicated that they are willing to move) were able to select more than one motive, or indeed “another motive” (not further specified, so not reported). Therefore, the percentages per column do not sum to 100%

The share mentioning Work is very comparable to what Coulter and Scott (2015) report for the UK, but Morrison and Clark (2011) report shares of about a third for New Zealand, whereas Niedomysl (2011) reports that 26% mention work, using a large-scale survey on Swedish migrants. Factors related to the dwelling are a less prevalent motive in these earlier contributions, and the relative importance of family and friends is generally higher. There are a number of conceivable explanations for these differences in the relative importance of migration motives. Country-specific factors might play a role, with size and accessibility influencing the need to be spatially mobile. Furthermore, some studies, such as Niedomysl (2011) cover actual migrants, whereas others pertain to prospective migrants (Coulter and Scott 2015)—as we do in this study. This is potentially important, as there are differences between motives in terms of the likelihood that they generate actual migration behaviour, with more specific motives generating a higher likelihood of actual migration (Coulter 2013a, b). Likewise, a respondent’s statement on what was the actual motive may change because of experiencing the move and its outcomes, after cognitive dissonance. A third potential cause lies in the fact that in this paper we do not exclude respondents that have moved or wish to move over a distance less than a certain threshold. Short distance moves are likely to be associated with residential motives (Thomas et al. 2019), and excluding them, as has been done in earlier contributions, affects the relative importance of Dwelling and Neighbourhood motives. Fourth, and most relevant to our objectives, the time dimension might be relevant. If people respond indeed to adverse economic circumstances by reducing mobility, or engage in mobility for different reasons, this might influence these statistics. In that light, it is interesting to observe that in our data, “Work” is relatively less important than “Dwelling”. It is also interesting to note that, as time progresses following the GFC, the incidence for all motives increases, apart from “Neighbourhood”. Respondents were allowed to indicate more than one motive. This indicates that, among those willing to move, and as the fallout of the GFC progressed, increasingly often a mix of motives appears relevant, rather than one main motive. This is a first indication of the heterogeneity of the impact of the GFC on desired mobility in our sample.

### General economic circumstances: impact of the global financial crisis across time and space

**Fig. 3:**
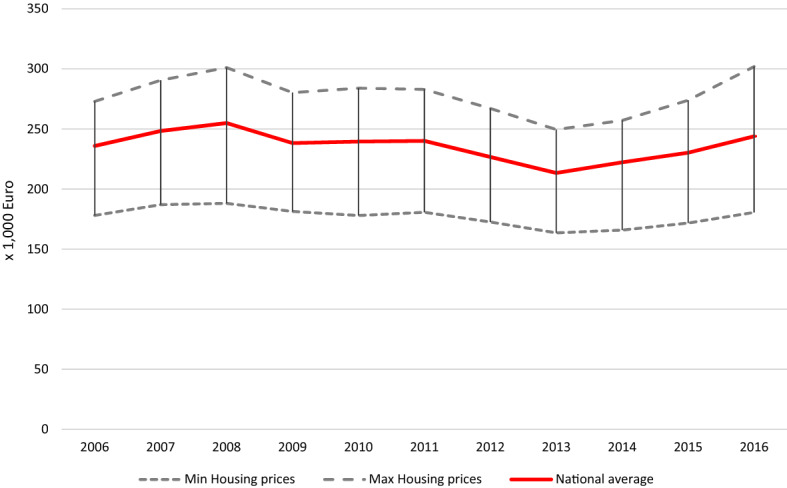
Average House Prices (transactions), Dutch NUTS 2 regions. Source: Statistics Netherlands

**Fig. 4:**
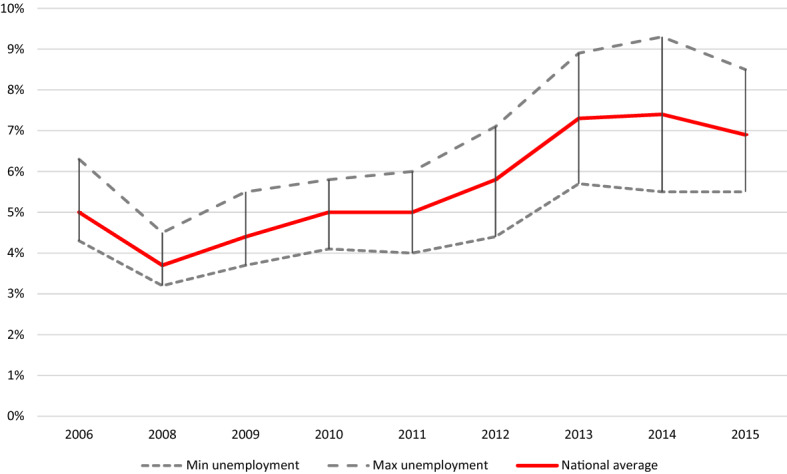
Unemployment rates, Dutch NUTS 2 regions. Source: Statistics Netherlands

As indicated above, in this paper, we measure the impact of the GFC using period dummies along with relative running indices of regional housing prices (based on transactions) and unemployment rates. In Fig. 3, we depict the national development in average housing price (central line) and the regional spread surrounding this national average (error bars and dashed lines, for NUTS2 regions) for the years during and following the GFC. Housing prices are, on average, highest in the provinces of Utrecht and Noord-Holland, and lowest in the northern province of Groningen. Furthermore, it becomes clear that, in terms of house prices, the GFC first led to stagnation, and then, with some delay, a decline in average house prices, followed by a slight recovery, for all NUTS 2 regions. The housing prices thus respond with some delay to the onset of the crisis in 2008. We also consider the regional unemployment rate, measured at the NUTS2 level, as an additional measure of the impact of the GFC (Fig. 4). The central line depicts the national unemployment rate, whereas the regional spread for Dutch NUTS2 regions is indicated using vertical bars and dashed lines. Typically, the more peripheral regions exhibit higher unemployment rates, but high rates are also found in more economically dense regions such as the province of Zuid-Holland. Here we observe that unemployment responded to the GFC also with some delay: rates were at their highest in 2013–2014.

### Other control variables

Some people prefer their current municipality because of local social networks, dependence on services such as a school or for other reasons. If so, the dummy *Wishes to stay in municipality* takes on a value of 1. This is a necessary control, as most people that prefer their current municipality also prefer the same type of neighbourhood. Nevertheless, 16% of our respondents desire to move up or down the urban hierarchy while staying in their current municipality. This is indeed possible, as within larger municipalities both neighbourhoods labelled as “Green-Centre” and “Rural” can be present, for example. Furthermore, through this variable we control for the distance of the intended move. This is essential too as local moves are often distinguished in the literature from migration, in terms of motives and distance traversed. We do not observe actual moves, and as such, we cannot directly control for actual distance traversed.

We control for characteristics of the dwelling currently occupied as well as those of the dwelling that is desired. WoON pays ample attention to the current type of residence and the preferred one. One question deals with the type of residence, for which we distinguish between “house”; “apartment” and “other” (such as a farm, home business; or a building with shared facilities). We also take into account whether people prefer to rent or own their residence, and what their current situation is in that respect.

The WoON survey collects demographic and other (economic) information about both the respondent and her/his family. We use the age of the respondent, whether the person is (self)employed, ethnicity (Dutch, Western or Non-Western as measured by location of birth), length of residence and the household disposable income. We apply a nested version of the original WoON household variables in our model. We simultaneously control for household composition, the presence of children and the age of the youngest child in the household, and the employment state of the partner. The category “other” refers to non-family households. The overall reference category is “Single Without Child”. We control for all of these factors, as they might influence willingness to move, the related motive, as well as the tendency to move up or down the urban hierarchy. Furthermore, we have also added regional fixed effects, for the NUTS 1 regions North, East, West and South, as well as for the four largest cities separately. These fixed effects are entered to pick up on any time-fixed characteristics of these areas.

## What determines the willingness to move up or down the urban hierarchy?

Before turning to our analysis of the relationship between economic circumstances that impact on the willingness to move up or down the urban hierarchy, we must first shed light on whether individuals indeed become more or less willing to move to begin with. Furthermore, as emanated from the literature, impacts of economic crises and their effects on (desired) mobility were found to be diverse and we analyse therefore whether adverse economic circumstances solely lead to a desire to move for work, or whether other motives come into play as well. All this will feed into our analysis of desired mobility across the urban hierarchy.

### Willingness to move

Table 2 shows the relative risk ratios that result from a binary logistic regression, executed in STATA, on the dependent variable *Willingness to move* (yes/no). Those who do not want to move are taken as the reference category. As described earlier, 27% of the respondents want to move within the next two years.

**Table 2:** Logit analysis of the willingness to move

	Willingness to move (1 = yes)
Currently lives in Central-urban (ref)	1
Currently lives in Sub-urban	0.987
Currently lives in Green-urban	0.892**
Currently lives in Village-centre	0.807***
Currently lives in Rural	0.664***
Natural log of duration of stay	1.205***
Currently lives in owned home (ref)	1
Currently lives in rented home	1.510***
Currently lives in house (ref)	1
Currently lives in apartment	1.466***
Currently lives in other	1.406***
Employed (ref)	1
Non-employed	1.018
Household income	1.002***
Single/no child (ref)	1
Couple/no child/working partner	0.821***
Couple/no child/partner not working	1.064***
Couple/child ≤ 11/working partner	0.606***
Couple/child ≤ 11/partner not working	0.896
Couple/child 12 ≥ Working partner	0.577***
Couple/child 12 ≥ partner not working	0.700***
Single parent/child ≤ 11	0.992
Single parent/child 12 ≥	1.043
Other	1.163***
Age	0.955***
Native (ref)	1
Non-western foreign	0.920***
Western foreign	1.051**
NUTS2 house price *t*/*t − *3 relative to NL	2.342
NUTS2 unemployment rate *t*/*t* − 3 relative to NL	0.947
Wave 1 (2008–2009) (ref)	1
Wave 2 (2011–2012)	1.067***
Wave 3 (2014–2015)	1.774***
Regional fixed effects	Yes
Observations	183,897
Pseudo-*R*2	0.0991

Exponentiated coefficients (odds ratios) reported; standard errors clustered at NUTS2 level

*Sources*: WoON 2008–2015 and Statistics Netherlands

^*^p < 0.10; **p < 0.05; ***p < 0.01

#### The willingness to move increases in times of economic hardship

We find that the GFC leaves its marks. Controlling for a rich set of personal, household and location characteristics, we find that the probability to indicate a willingness to move increased in the years following the GFC, when house prices were at their lowest and unemployment was at its highest. Compared to our first wave, covering 2008–2009, respondents in the second and third wave were 6.7% and 77.4% more likely to indicate that there is a discrepancy between their current residential situation and the desired residential situation. We find no additional effects from the relative development in the regional house prices and unemployment rates. This implies that adverse circumstances appear to increase the willingness to move. Although the literature suggests lower levels of migrations during periods of crisis (e.g. Cooke 2013), our results suggest that people are actually more willing to move although that desire might be hampered by hurdles brought up by the economic stagnation.

#### Results for other control variables

Starting with the personal and household characteristics, Table 2 shows that, compared to those that own their home, renters are keener to find a new place, as are people who live in an apartment or another type of dwelling when we compare them to those living in a house. Renting and living in apartments is more prevalent in urban neighbourhoods, but we find this result after controlling for the nature of the neighbourhood. This residential environment plays an important role as well: people living in the Central-Urban and Sub-Urban areas have a higher probability (risk) of wanting to move than people currently living further down the urban hierarchy. This confirms the descriptive patterns reported in Fig. 2. Compared to natives, non-Western foreigners are less willing to move, but Western foreigners are slightly more willing to move. Willingness to move increases with income, but there is no relation with personal employment status—as opposed to the role of the general economic circumstances. In addition, willingness to move decreases with age. Interestingly, those who have been living in their current home for a long time have a higher probability of wanting to move. This appears at odds with findings in the literature on inertia: actual migration probabilities decline with longer durations of stay, as discussed in Sect. 2. However, Age and Duration of Stay are strongly positively correlated. After controlling for Age, Duration of Stay captures increasing residential dissonance. When looking at the household composition, it is interesting to see that Couples with an employed partner appear less likely to be willing to move, compared to similar households with a non-working partner. In particular, childless couples with a non-working partner are relatively willing to move, as are childless Singles.

### Motives for migration

In Table 3, we report the results of six separate binary logistic regression models, each with a specific motive as an outcome. If the motive was mentioned, the dummy outcome variable is coded "1". We again control for a rich set of personal and regional characteristics, selecting only those characteristics that are known before the prospective move. Those who do not wish or intend to move are not included in this estimation sample.

**Table 3:** Logit analysis of the motives for moving

	Health	Study	Work	Dwelling	Neighbourhood	Family/friends
Currently lives in Central-urban (ref)	1	1	1	1	1	1
Currently lives in Sub-urban	1.022	0.818***	0.836***	0.947	1.469***	1.174***
Currently lives in Green-urban	1.012	0.939	0.951	0.838***	1.072	1.220***
Currently lives in Village-centre	0.952	0.808*	0.785***	0.828***	0.915	1.325***
Currently lives in Rural	0.976	1.019	0.972	0.612***	0.862	1.585***
Employed (ref)	1	1	1	1	1	1
Not employed	2.123***	1.698***	0.700***	0.923***	0.951*	1.173***
Household income	0.991***	0.971***	1.005***	0.999*	0.999*	0.999
Single/no child (ref)	1	1	1	1	1	1
Couple/no child/working partner	1.150**	0.288***	0.604***	1.208***	1.197***	0.775***
Couple/no child/partner not working	1.615***	0.983	0.497***	0.949**	0.855***	0.749***
Couple/child ≤ 11/working partner	0.606***	0.482***	0.496***	1.620***	1.407***	0.721***
Couple/child ≤ 11/partner not working	1.241	0.486***	0.619***	1.641***	1.446***	0.663***
Couple/child 12 ≥ /working partner	1.110	4.188***	0.780***	1.060	1.560***	0.420***
Couple/child 12 ≥ /partner not working	1.889***	3.516***	0.675***	1.126	1.133	0.457***
Single parent/child ≤ 11	1.210*	0.441***	0.328***	1.292***	1.703***	0.924
Single parent/child 12 ≥	1.009	2.172***	0.607***	1.204***	1.231***	0.760***
Other	1.179	2.655***	0.890*	0.551***	0.777***	0.776***
Age	1.080***	0.871***	0.955***	0.979***	0.995***	1.000
Native (ref)	1	1	1	1	1	1
Non-western foreign	1.439***	0.802	0.641***	1.528***	0.807***	1.022
Western foreign	0.875*	1.103	1.321***	1.005	0.903**	1.104**
NUTS2 house price *t*/*t* − 3 relative to NL	0.416	0.107	1.089	1.089	3.179	0.378
NUTS2 unemployment rate *t*/*t* − 3 relative to NL	0.421***	0.546	0.511**	1.240	1.461	0.652
Wave 1 (2008–2009) (ref)	1	1	1	1	1	1
Wave 2 (2011–2012)	1.126**	1.406***	1.157***	1.084*	1.066	1.271***
Wave 3 (2014–2015)	1.604***	1.537***	1.238***	1.647***	1.213***	1.501***
Regional fixed effects	YES	YES	YES	YES	YES	YES
Observations	37,389	37,389	37,389	37,389	37,389	37,389
Pseudo-*R*2	0.2969	0.3243	0.0899	0.0507	0.0222	0.0133

Exponentiated coefficients (odds ratios) reported; standard errors clustered at NUTS2 level

*Sources*: WoON 2008–2015 and Statistics Netherlands

^*^p < 0.10; **p < 0.05; ***p < 0.01

#### Adverse economic circumstances stimulate mobility for a wide variety of motives

We find a significant increase in the likelihood of mentioning all motives, as the fallout of the GFC took hold over time. Even though it is not possible to directly compare logit coefficients across models, generally speaking the results suggest that the willingness to move for Health-related reasons and reasons related to the Dwelling increased relatively strongly, closely followed by wanting to move for Study related reasons, or moving closer to Family and Friends. Moreover, in NUTS2 regions where the increase in the unemployment rate was relatively strong, the willingness to move for Health reasons and for Work was less strong than the general trend. From this, we conclude that the impact of the GFC is such that households not only become more willing to move, but they wish to do so for an increasingly wide variety, and combination, of motives. It therefore stands to reason to expect that economic crises do not equivocally lead to moves towards the opportunity-rich urban areas. Depending on the exact impact, as signalled by motive, other desires may emerge as well. Crisis-led mobility is certainly not just about moving for Work-related reasons. Our results suggest that the desire to move for Work-related reasons does increase, but particularly so in regions that are relatively less affected. It is perhaps concerning to note that in regions which bear more of the crisis unemployment fallout, the willingness to move for Work does not increase as strongly, suggesting worker discouragement.

#### Results for control variables

The degree of urbanity of the current neighbourhood does not influence the willingness to move for Health. Summarising the other results, and noting that not all coefficients are significant: compared to those living in Central-Urban areas, those living further down the urban hierarchy tend to be less likely to want to move for Study, Work and reasons related to the Dwelling. However, they are more likely to express a desire to move closer to Family and Friends. Furthermore, compared to Central-Urban, residents of Sub-Urban appear to wish a change of neighbourhood. This suggests that unmet demand for (particular types of) housing is highest in cities, but, rather more surprisingly, locations further down the urban hierarchy offer ample access to healthcare, study and work opportunities. Likewise, the results suggest that cities and towns provide better opportunities to live close to family and friends. This points again to a large diversity in (unmet) needs, but likewise to a role of urban areas that goes beyond mere opportunities to work. Unemployed respondents are more likely to want to move for health, study and family and friends, but less likely to mention work, the dwelling or the neighbourhood. This is a rather surprising result in itself, but perhaps fitting in the Dutch and European context where individuals are typically less likely to want to move for work than, for example, the USA (Cooke 2013). Wanting to move for work does become more likely with increasing levels of income. Considering the household characteristics, singles without children are found to be most likely to want to move for work and family/friends and—generally speaking—least likely to desire a move for reasons related to the neighbourhood or the dwelling.

### Moving up or down the hierarchy

On the back of the supporting analyses reported in the previous sections, we now turn to our main question on the relationship between the general economic circumstances and the desire to move up or down the urban hierarchy. Table 4 presents three logit models, with “up”, “same” and “down” as the outcomes under study. Those who do not wish or intend to move are not included in the estimation sample. For the “up” and “down” models, “same” is the reference outcome. For “same”, “up or down” is chosen as the reference category. Valid *N* for these models thus partly overlaps. Further to the control variables used in the other models, we now also include controls for the stated desires with respect to the dwelling, and include the motive in our analysis.

**Table 4:** Logit analysis of the discrepancy between current and desired degree of urbanity of the neighbourhood

	Up	Same	Down
Motive to move Health	0.920**	1.031	1.008
Motive to move Study	1.103	1.205	0.636***
Motive to move Work	0.980	1.132***	0.830***
Motive to move Dwelling	0.917*	1.066*	0.949
Motive to move Neighbourhood	1.649***	0.540***	1.880***
Motive to move Family/friends	1.293***	0.870**	1.035
Currently lives in Central-urban (ref in “Same” and “Down”)		1	1
Currently lives in Sub-urban (ref in “Up”)	1	1.386***	0.399***
Currently lives in Green-urban	3.445***	1.075	0.222***
Currently lives in Village-centre	1.746***	2.110***	0.139***
Currently lives in Rural	4.478***	1.671***	
Natural log of duration of stay	0.914**	1.042	1.002
Currently lives in House (ref)	1	1	1
Currently lives in apartment	0.936**	1.124***	0.847***
Currently lives in other	1.536***	0.867*	0.885*
Desires house (ref)	1	1	1
Desires apartment	2.016***	0.961	0.622***
Desires other	1.112	0.739***	1.580***
Currently lives in owned home (ref)	1	1	1
Currently lives in rented home	1.025	1.044	0.933***
Desires owned home (ref)	1	1	1
Desires rented home	0.957	1.132***	0.825***
Wish to remain in same municipality (yes = 1)	0.238***	4.672***	0.202***
Employed (ref)	1	1	1
Non-employed	0.959	1.048**	0.944
Household income	1.002**	0.999	1.000
Single/no child (ref)	1	1	1
Couple/no child/working partner	0.822**	1.045	1.041
Couple/no child/partner not working	0.822**	1.109*	0.926
Couple/child ≤ 11/working partner	0.545***	1.324***	0.937
Couple/child ≤ 11/partner not working	0.574***	1.359***	0.884
Couple/child 12 ≥ /working partner	0.748***	1.064	1.131**
Couple/child 12 ≥ /partner not working	0.583***	1.343***	0.901
Single parent/child ≤ 11	0.706***	1.222***	0.923
Single parent/child 12 ≥	0.995	1.024	0.947
Other	0.914	1.167***	0.814***
Age	0.993***	1.003*	1.001
Native (ref)	1	1	1
Non-western foreign	1.031	1.127***	0.801***
Western foreign	1.024	1.038	0.908*
NUTS2 house price *t*/*t* − 3 relative to NL	1.479	2.680	0.0648*
NUTS2 unemployment rate *t*/*t* − 3 relative to NL	1.913**	0.802	0.824
Wave 1 (2008–2009) (ref)	1	1	1
Wave 2 (2011–2012)	0.857**	0.954	1.213***
Wave 3 (2014–2015)	0.998	0.895	1.226***
Regional fixed effects	YES	YES	YES
Observations	28,640	36,220	30,680
Pseudo-*R*2	0.1523	0.1246	0.1648

Exponentiated coefficients (odds ratios) reported; standard errors clustered at NUTS2 level

*Sources*: WoON 2008–2015 and Statistics Netherlands

^*^p < 0.10; **p < 0.05; ***p < 0.01

#### A rural refuge in volatile and uncertain times?

We find that moving up the urban hierarchy was desired less in the first years following the GFC, but not when the negative fallout was at its peak in the later years. However, in regions with relatively strong increases in the unemployment rate, wanting moving up the urban hierarchy was more likely. Our earlier insights from Tables 2 and 3 suggest that this intention to move out of such affected reasons is fuelled by other motives than merely finding work. In fact, expressing a desire to move down the urban hierarchy was more likely throughout the period following the GFC, following Table 4. Here we see that the overall finding, up to this point, of an increased desire to move, for an increasingly diverse combination of motives, translates spatially in a move down the urban hierarchy, rather than up. In regions which experienced a relatively strong decline in house prices, people are even more likely to want to move down the urban hierarchy. It appears that moving down the urban hierarchy is seen as a viable coping strategy if the household is vulnerable to price changes, perhaps related to what was noted above regarding the apparent quality of access to jobs and study, and dwellings in these areas.

In terms of motives to move, we might have expected to see that work and perhaps study related motives would lead to a higher likelihood of moving up the urban hierarchy. However, instead we find that a desire for moving up is associated with motives linked to the neighbourhood and moving towards the support networks of family and friends. Moving for work is associated with wanting to move to similar areas. Moving for study or work is related to a lower likelihood of moving down the urban hierarchy but is fuelled once more by a desire for a change of neighbourhood characteristics. The tendency to move down the hierarchy in times of economic hardship, as noted above, is not directly related to a work motive. So, here too we see a nuanced response.

#### Results for other control variables

Although 16% of the respondents is actually looking for a different environment within their current municipality, the odds are almost five times as large that people who want to move along the urban hierarchy intend to move to another municipality. Considering the degree of urbanity of the current residential neighbourhood, compared to those living in suburban areas, indeed all the other categories are more likely to want to move up the hierarchy. The relation is not consistently increasing, however: inhabitants of “Village-Centre” are, relatively speaking, less likely to desire a move up the hierarchy than their neighbours in the Rural and Green-Urban. In the “same” analysis, together to those in Central-Urban, potential migrants in Green-Urban are the least likely to desire a similar neighbourhood. Inhabitants of Central-Urban neighbourhoods are by far the most likely to express a desire to move down the urban hierarchy. This desire, a latent demand for (more) rural living perhaps, decreases with decreasing levels of urbanity.

The duration of stay influences the likelihood to desire a move up the urban hierarchy negatively. Older respondents are less likely to desire a move up the urban hierarchy as well. Those with a non-western background do not desire neighbourhoods which are very different from their current location, and in particular do not desire to move down the urban hierarchy.

Relative to singles without children, all couples and the single parents with a child younger than 11 years old are less likely to desire a move up the urban hierarchy. Couples with young children desire a change to a similar neighbourhood instead, irrespective of the employment status of the partner. Couples with children past the elementary school ages and with a working partner prefer a move down the urban hierarchy, towards the rural. Apart from this, the employment status of the partner does not appear to play a very pronounced role. Dual earner households have more complex locational puzzles to solve, but often also have the financial resources to organise and afford commuting. Yet, we also find that higher household income is associated with a desire to move up the urban hierarchy.

Another reason for wanting to move is the type of dwelling that is preferred. A desire to move into renting is related to a preference for similar neighbourhoods. Currently living in a rented home as well as desiring one leads to a lower likelihood of wanting to move down the urban hierarchy. People who are currently living in an apartment are more likely to search for similar surroundings than residents of houses, while residents of a house are more likely to want to move down the urban hierarchy. In addition, if people desire an apartment, the odds are twice as large that they want to move to a more urban area compared to family-home residents. Not surprisingly, respondents that desire to live in an “other” type of dwelling, such as a farm, a residence with shared space(s) or a home business, often prefer a more rural environment where such residences are typically located.

## Conclusions and discussion

In this paper, we set out to investigate the relationship between the adverse economic circumstances following the Global Financial Crisis (GFC) and the desire of Dutch households to move up or down the urban hierarchy. We argue that, in particular in economically testing times, focussing on stated mobility intentions (in explicit relation to the current residential location) is to be preferred to measuring revealed mobility, given that not all households will be able to effectuate their desires and use mobility in response to crises. Measuring such unmet demand sheds light on the heterogeneous impact of economic crises on households, resulting in needs which may stretch beyond the immediate opportunities to engage in spatial job search.

We applied three consecutive waves of the Dutch Housing Demand Survey (WoON) in a repeated cross-section setting, with data collected at the time of the GFC and its aftermath. This dataset allowed us to measure household desired destinations in the years following the onset of the GFC, whilst controlling for all the standard time, life course and regional factors emanating from the literature.

We find that households desire to move down the urban hierarchy during the volatile and uncertain periods following the GFC. This is a surprising result, considering that urban areas are generally considered more opportunity rich.

In order to uncover the mechanism driving this result, we considered the impact of the economic circumstances on the general willingness to move. Furthermore, and also fuelled by the heterogeneous results emerging from the relevant literature, we have investigated whether adverse economic circumstances indeed lead to a greater motive to move for economic reasons, or whether in fact other motives for mobility become more important which might serve to explain this result.

We find that willingness to move down the urban hierarchy increases when decline in housing prices is more prominent, and when unemployment levels are high. Looking at motives for migration, it appears that this increased willingness to move is only partially fuelled by a desire to move for work. Rather, we found that moving for health, education, reasons related to the dwelling, and towards family and friends, became relatively more important during the negative aftermath of the GFC. This suggests a heterogeneous impact on households of the GFC and helps to better understand the mechanism behind the expressed desire to move down the urban hierarchy in such circumstances.

Moreover, the more rural the current residential location, the lower the probability of wanting to move for the dwelling, which suggests that in particular in the urban areas there is an unmet demand for specific types of housing. Work and study related motives do not lead to a higher likelihood of moving up the urban hierarchy. However, instead we find that moving up the urban hierarchy is associated with motives linked to the neighbourhood and moving towards family and friends. In particular, the latter result, a desire to move towards family and friends in cities, could point to a whole different concept of support and opportunity in cities: networks appear important rather than a thick labour market, or perhaps a means to enter an otherwise tough residential market. Moving for work is associated with wanting to move to similar areas.

All in all, our results suggest that immobility in times of crisis, as reported elsewhere, is not necessarily the desired response to a crisis. Our results suggest that low and declining revealed mobility rates in times of crisis, reported in the literature, are possibly the result of constraints to moving, in particular into urban areas, rather than a lack of willingness to move or openness to other destinations. Well-developed forms of transport, or the application of ICT is necessary to remain in touch with these labour markets. Likewise, the impact of the GFC is found to be diverse, and to generate a willingness to move for a wide variety of reasons, and, consequently, to a wide variety of destinations. Access to support networks and characteristics of the dwelling appear as relevant as more economic considerations. Here we can draw important lessons, considering the impact of current and future crises. The current Covid-19 pandemic, whilst its full economic impact is yet to be felt, has already generated a demand for in- and outdoor space to facilitate working from home. An, albeit modest, Covid-19 fuelled increase in moves towards destination outside the economic core areas in the Netherlands has already been reported by Statistics Netherlands (Statistics Netherlands 2021). It could very well be that housing market constraints need to be cleared both up and down the urban hierarchy.

A limitation of the current study is the cross-sectional nature of the data. Panel data would have put us in a better position to deal with unobserved heterogeneity. Likewise, we were not able to include data on households in the period leading up to the GFC. We have, however, managed to capture major dynamics during and following the GFC. Future research could further capitalise on this, and consider whether the heterogeneity in stated intentions translates into heterogeneity in revealed outcomes as our study suggests.

## Footnotes

1 The relative running indices are calculated using (*X*_*j,t*_*/X*_*j,t*−3_)/(*X*_*N,t*_/*X*_*N,t*−3_), for NUTS2 regions (i.e. Dutch provinces) *j* and year *t*, relative to the index for the Netherlands as a whole (*N*).

2 Because this imputation method changed, we could not use the 2006 wave in our analysis. It is therefore not possible to include the period leading up to the GFC.

3 These percentages do not sum to 100%, as the populations at risk differ between these separate analyses. The sample at risk of moving up the urban hierarchy excludes those already living in a central urban environment. Similarly, those at risk of moving down do not include people living in rural areas.
